# Supplementary material for: Structured Docosahexaenoic Acid (DHA) Enhances Motility and Promotes the Antioxidant Capacity of Aged C. elegans
Source: Cells. 2023 Jul 26;12(15):1932. doi: 10.3390/cells12151932 (PMC10417004; doi:10.3390/cells12151932)
Supplement: Supplementary file 1 [file cells-12-01932-s001.zip › cells-2505969-supplementary.pdf]

Supplementary material

Structured Docosahexaenoic Acid (DHA) enhances healthspan and promotes antioxidant metabolism of aged *C. elegans*

Ignasi Mora [1*], Alejandra Pérez-Santamaria [2], Julia Tortajada-Pérez [3], Rafael Vázquez-Manrique [4], Lluís Arola [5] and Francesc Puiggròs [6*]

1 Brudy Technology S.L., 08006 Barcelona, Spain; cultivos1@brudylab.com
2 Eurecat, Centre Tecnològic de Catalunya, Nutrition and Health Unit, 43204 Reus, Spain; alejandra.perez@estudiants.urv.cat
3 Laboratory of Molecular, Cellular and Genomic Biomedicine, Instituto de Investigación Sanitaria La Fe, 46026 Valencia, Spain; julia_tortajada@iislafe.es (J.T.-P.); rafael_vazquez@iislafe.es (R.P. V.-M.)
4 Joint Unit for Rare Diseases IIS La Fe-CIPF, 46012 Valencia, Spain
5 Centro de Investigación Biomédica en Red de Enfermedades Raras (CIBERER), 28029 Madrid, Spain
6 Nutrigenomics Research Group, Departament de Bioquímica i Biotecnologia, Universitat Rovira i Virgili, 43007 Tarragona, Spain; lluis.arola@urv.cat
7 Eurecat, Centre Tecnològic de Catalunya, Biotechnology Area, 43204 Reus, Spain; francesc.puiggros@eurecat.org

*Correspondence: cultivos1@brudylab.com; francesc.puiggros@eurecat.org

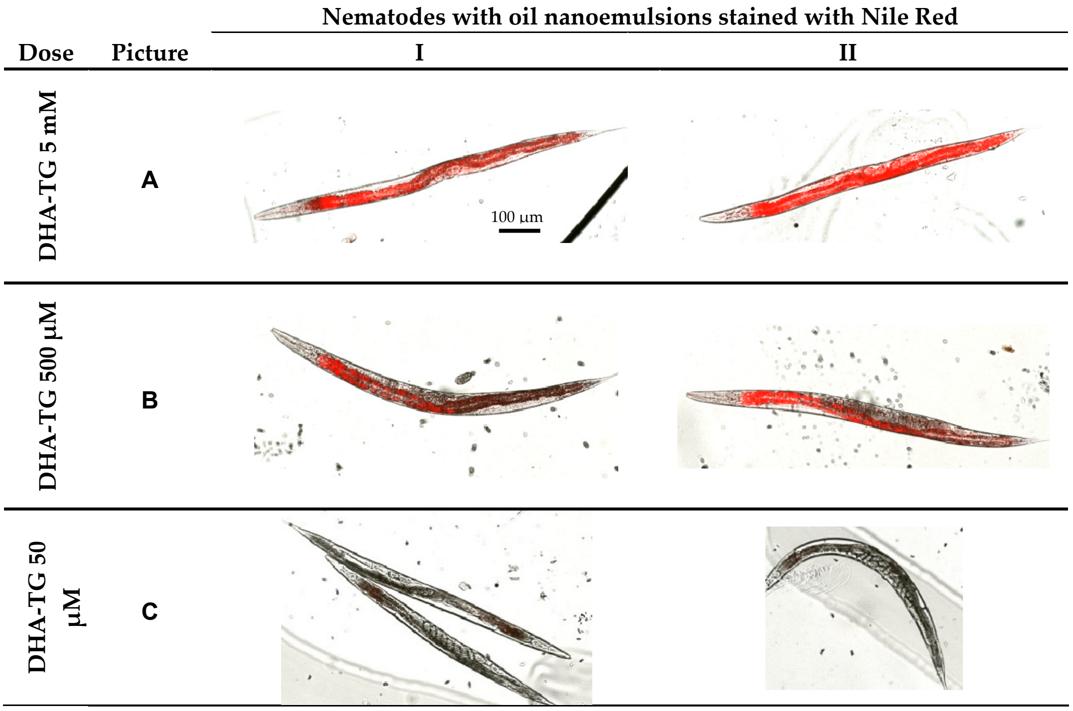

Figure S1. Confocal images of worms grown on agar containing nanoemulsions of DHA-TG oil stained with Nile Red. Images A-C show decreasing concentrations of DHA. Pictures I and II show different nematodes treated with the same concentration on DHA. Green arrows at the dark pictures C (i) are pointing nematode's fluorescence in the pharynx. All the pictures have the same scale as picture A (I).

Supplementary material

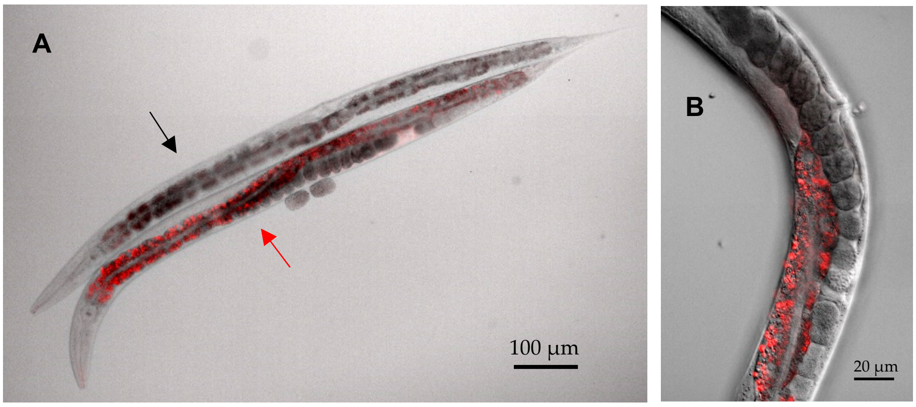

Figure S2. Confocal images of worms grown on agar containing nanoemulsions of DHA-TG oil stained with Nile Red. (A) Black arrow points one nematode treated with DHA-TG nanoemulsion; red arrow points a nematode treated with nanoemulsion containing Nile Red. Both at the same DHA-TG concentration 500 μM. (B) picture showing DHA-TG nanoemulsions around intestinal tract, not in gonads or eggs.

Supplementary material

Table S1. Primers used for the amplification of the cDNA

Gene (Wormbase accession nº)	Encoding	Primer sequence (5' → 3')
sod-3 (C08A9.1)	Mitochondrial Superoxide dismutase	F- GGCTAAGGATGGTGGAGAAC<br>R- ACAGGTGGCGATCTTCAAG
gst-7 (F11G11.2)	Glutathione transferase	F- GACAGCTTCCACTCCTTGAA<br>R- GCACACTTTCCATTGATTCC
gcs-1 (F37B12.2)	γ-GlutamylCysteine Synthetase	F- AATCGATTCCTTTGGAGACC<br>R- ATGTTTGCCTCGACAATGTT
skn-1 (T19E7.2)	Nuclear factor erythroid 2-related factor 2 (NRF2)	F- GTTCCCAACATCCAACTACG<br>R- TGGAGTCTGACCAGTGGATT
ctl-1 (Y54G11A.6)	Catalase	F- AATGGATACGGAGCGCATAC<br>R- TCCTGTTCAGCACCATCTTG
sir 2.1 (R11A8.4)	Sirtuin	F- TGGCTGACGATTCGATGGAT<br>R- ATGAGCAGAAATCGCGACAC
aak-2 (T01C8.1)	AMP-Activated Kinase (AMPK)	F- TGCTTCACCATATGCTCTGC<br>R- GTGGATCATCTCCCAGCAAT
daf-16 (R13H8.1)	Forkhead box (FOXO)	F- TCAGGGATAAGGGAGATTCG<br>R- CAGATTGTGACGGATCGAGTT
